# Role of nitric oxide and cGMP in the modulation of vascular contraction induced by angiotensin II and Bay K8644 during ischemia/reperfusion

**DOI:** 10.3892/etm.2012.846

**Published:** 2012-11-30

**Authors:** KATARZYNA SZADUJKIS-SZADURSKA, GRZEGORZ GRZESK, LESZEK SZADUJKIS-SZADURSKI, MARTA GAJDUS, GRZEGORZ MATUSIAK

**Affiliations:** Department of Pharmacology and Therapeutics, Collegium Medicum Nicolaus Copernicus University, Bydgoszcz 85-094, Poland

**Keywords:** angiotensin II, nitric oxide, Bay K8644, contraction, ischemia/reperfusion

## Abstract

Vascular smooth muscle tone changes under the influence of numerous contracting and relaxing factors. The purpose of the present study was to determine the modulating effect of ischemia and reperfusion (I/R) on contraction triggered by angiotensin II (ANG II) and Bay K8644 as well as to investigate the importance of nitric oxide (NO) and cGMP in these reactions. Experiments were performed on isolated and perfused Wistar rat tail arteries. The contraction triggered by ANG II and Bay K8644 with the use of intracellular (in calcium-free physiological salt solution; FPSS) and extracellular (in physiological salt solution; PSS) pools of calcium ions after I/R and in the presence of sodium nitroprusside (SNP), ^8^Br-cGMP, an endothelial NO synthase (NOSe) inhibitor (L-NG-nitroarginine methyl ester; L-NAME) or ODQ [an inhibitor of soluble guanylyl cyclase (GC)] was evaluated. ANG II triggered contraction in FPSS and PSS, but Bay K8644 only in PSS. Ischemia reduced and reperfusion intensified the response of the artery to ANG II, but did not change the action of Bay K8644. SNP and ^8^Br-cGMP reduced the response of the vessels to ANG II and did not change the modulating effect of ischemia, but reduced the intensifying action of reperfusion on contraction caused by the presence of ANG II. SNP lowered the action of Bay K8644 in PSS. In PSS, L-NAME and ODQ intensified the action of ANG II, eliminating the reducing effect of ischemia on the contraction caused by ANG II, but did not influence the intensifying reaction caused by reperfusion. L-NAME and ODQ did not influence the action of Bay K8644. I/R modulated the contraction of arteries triggered by ANG II, but did not influence the response to Bay K8644. The intra- and extracellular pools of calcium ions mediate the action of ANG II, but Bay K8644 stimulated contraction only with participation of calcium ions flowing into the cell. Control of the vascular smooth muscle tone associated with the action of NO and cGMP is subject to modulation under conditions of I/R.

## Introduction

The contractibility of blood vessels depends on their normal structure and the availability of calcium ions; it changes under the influence of a number of contracting [e.g., angiotensin II (ANG II) and endothelin-1] and relaxing [e.g., nitric oxide (NO) and prostacyclin] factors, which control the activities of various pathways of intracellular and intercellular signaling (1–3). The endothelium lines the inside of the vessel and is not only an integral part of the vessel structure, but also actively participates in its normal functioning. Endothelial cells produce substances which regulate contraction-relaxation activity, but the action of the internal layer of the vessel is subject to the influence of various factors, acting via receptors (1,4). Smooth muscle contraction, stimulated by a number of physiological factors, may take place with the participation of calcium ions released from the intracellular reserve, which flow from the extracellular space through the channels in the cellular membrane. However, an excessive increase in calcium ion concentration may promote cell death as a result of apoptosis, ischemia/reperfusion (I/R) and excitotoxicity (5–7). The smooth muscle is also important in the pathogenesis of vascular diseases, including sclerosis, arterial hypertension and restenosis (8,9).

I/R and oxidative stress are pathological phenomena, which may change the reactivity of the vessels and modulate the effect of substances which control the vascular smooth muscle tone (10,11). Tissue damage caused by hypoxia is mediated by mechanisms associated with oxygen (reactive oxygen species, ROS) and nitrogen (reactive nitrogen species, RNS) released in large amounts after reperfusion, which promote inflammatory processes, cell death and organ failure. Such phenomena are responsible for the failure of bypass graft surgery and organ transplantation surgery, as well as complications of myocardial, cerebral and renal hypoxia syndromes (12–15).

The purpose of the present study was to establish the modulating effect of I/R on contraction triggered by ANG II (an agonist of the metabotropic AT1 receptor) and Bay K8644 (an agonist of calcium channels located in the cellular membrane) as well as to investigate the role of the signaling pathway associated with NO and cGMP in these reactions.

## Material and methods

### Preparation of arteries

In the experiment, Guiding Principles for the Care and Use of Animals in the Field of Physiological Sciences as well as specific national laws were followed. The Ethical Committee for the Affairs of Experiments on Animals in Bydgoszcz approved the experiments undertaken (No. 1/2008-4). All reagents were purchased from Sigma-Aldrich (Poland, Poznań). Studies were performed on isolated and perfused Wistar rats' tail arteries. Animals, with body weights ranging from 250 to 350 g, were anesthetized with urethane administered intraperitoneally at a dose of 120 mg/kg of body weight. In order to establish the effect of I/R on the reactivity of the vascular smooth muscle triggered by ANG II (30 nM/l) and Bay K8644 (30 *μ*M/l), a clamp was placed on the proximal segment of the prepared artery for 30 or 60 min and the artery was then removed. Then, a cannula was inserted into the proximal segment of the detached fragment of the rat's caudal artery measuring 2.5–3 cm in length, which was subsequently connected to the perfusion system and the equipment to allow constant measurement and recording of the perfusion pressure. The distal end of the prepared artery was loaded with a weight of 500 mg, and the preparation was placed in a vertical position in a thermostatic dish intended for isolated organs with a volume of 20 ml, oxygenated with saline at a temperature of 37°C. The flow of the perfusion fluid was gradually increased until it reached 1 ml/min. During the next stage, the contraction of the arteries was evaluated after 30, 60 and 120 min of reperfusion.

### Assessment of role of calcium ion pools

In order to evaluate the involvement of intracellular and extracellular calcium ion pools in reactions triggered by the studied agonists under control conditions, following I/R and in the presence of sodium nitroprusside (SNP, 100 *μ*M/l) as a donor of NO, ^8^Br-cGMP (100 *μ*M/l), an endothelial NO synthase (NOSe) inhibitor (L-NG-nitroarginine methyl ester; L-NAME, 300 *μ*M/l) or ODQ [inhibitor of soluble guanylyl cyclase (GC), 100 *μ*M/l], the experiments were conducted using two types of Krebs fluid: i) Fluid without Ca^2+^-EGTA [Krebs (no calcium); calcium-free physiological salt solution (FPSS)], with the following composition: NaCl (71.8 mM/l), KCl (4.7 mM/l), NaHCO_3_ (28.4 mM/l), MgSO_4_ (2.4 mM/l), KH_2_PO_4_ (1.2 mM/l) and glucose (11.1 mM/l) with the addition of EGTA (30 *μ*M/l); ii) Fluid with Ca^2+^-EGTA [Krebs (normal); physiological salt solution (PSS)] with the the same composition as FPSS with CaCl_2_ (1.7 mM/l), after emptying the intracellular pool of calcium ions.

Evidence for the vessel contraction in the conducted experiments included an increase in pressure of the perfusate in an experimental system, at a preset flow of the perfusion fluid (∼1 ml/min).

### Statistical analysis

Results are presented as average values and standard deviation. Statistical differences were evaluated using the Student's t-test. P<0.05 was considered to indicate a statistically significant result. Calculations were conducted with Statistica 6.0PL software.

## Results

### Effects of ANG II and Bay K8644

**Figure 1.**
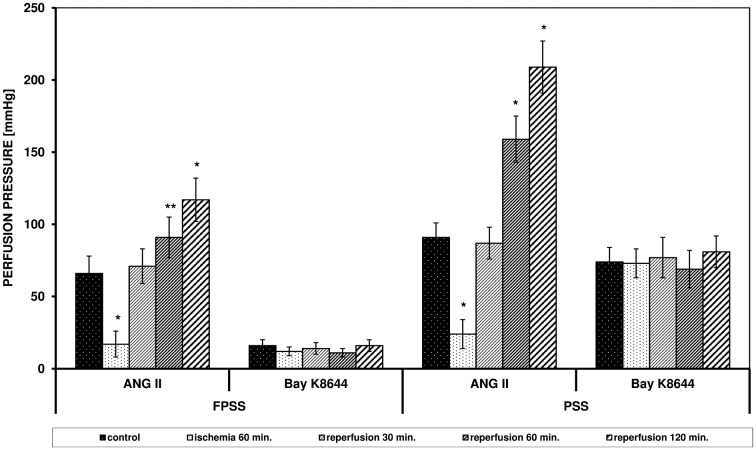
Effect of 60 min of ischemia and 30, 60 and 120 min of reperfusion on response of the arteries to ANG II (30 nM/l) and Bay K8644 (30 *μ*M/l) in FPSS and PSS; (mean ± SE, n=12); ^*^P<0.0001 vs. control; ^**^0.05>P>0.0001 vs. control. ANG II, angiotensin II; PSS, physiological salt solution; FPSS, calcium-free PSS.

ANG II triggered an increase in perfusion pressure in FPSS and PSS, but the values were higher in PSS (Fig. 1). After 60 min of ischemia, a reduced response of the arteries to ANG II was observed, but after 60 and 120 min of reperfusion, the maximum effects were significantly elevated in FPSS and PSS (in PSS after 60 and 120 min P<0.0001 vs. control; in FPSS after 60 min of reperfusion 0.05>P>0.0001). Bay K8644 triggered contraction only with the use of an extracellular pool of calcium ions, which, in contrast to the experiments with ANG II, was not modulated by I/R. The obtained values of reperfusion pressure triggered by ANG II and Bay K8644 following I/R in FPSS and PSS are presented in Fig. 1.

### Effects of SNP and ^8^Br-cGMP

**Figure 2.**
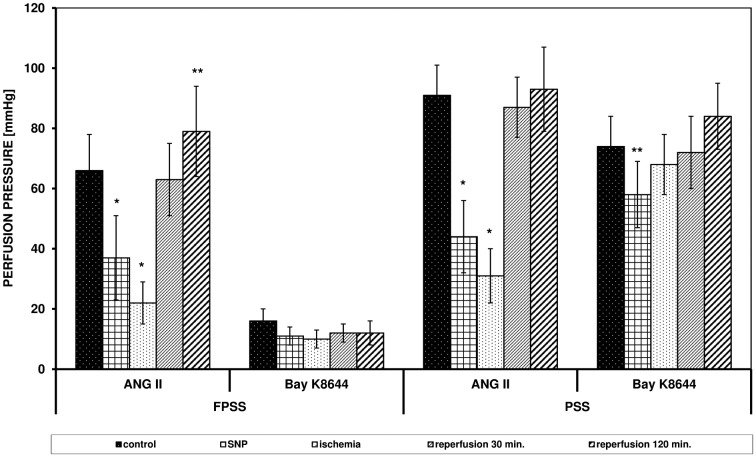
Effect of 60 min ischemia and 30 and 120 min of reperfusion on response of the arteries to ANG II (30 nM/l) and Bay K8644 (30 *μ*M/l) in the presence of SNP (100 *μ*M/l), in FPSS and PSS; (mean ± SE, n=12); ^*^P<0.0001 vs. control; ^**^0.05>P>0.0001 vs. control. ANG II, angiotensin II; SNP, sodium nitroprusside; PSS, physiological salt solution; FPSS, calcium-free PSS.

The addition of SNP (100 *μ*M/l) reduced the control response of the arteries to ANG II with involvement of intra- and extracellular pools of calcium ions and reduced the contraction triggered by Bay K8644 in PSS (Fig. 2).

SNP did not alter the effect of ischemia on contraction triggered by ANG II. In FPSS, the perfusion pressure was 22 mmHg (vs. 17 mmHg without SNP, P=0.24), and in PSS it was 31 mmHg (vs. 24 mmHg without SNP, P= 0.16). The presence of SNP significantly reduced the intensifying effect of reperfusion on the response of the arteries triggered by ANG II. In FPSS, the pressure after 120 min of reperfusion was 79 mmHg (vs. 117 mmHg without SNP, P=0.0002), and in PSS it was 93 mmHg (vs. 209 mmHg without SNP, P<0.0001). SNP reduced the contraction triggered by Bay K8644 in PSS (P= 0.0088).

**Figure 3.**
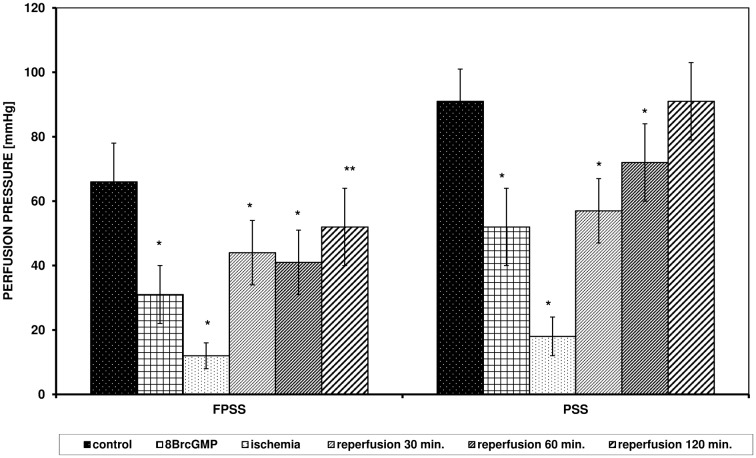
Effect of 60 min of ischemia and 30, 60 and 120 min of reperfusion on response of the arteries to ANG II (30 nM/l) in the presence of ^8^Br-cGMP (100 *μ*M/l), in FPSS and PSS; (mean ± SE, n=12); ^*^P<0.0001 vs. control; ^**^0.05>P>0.0001 vs. control. ANG II, angiotensin II; PSS, physiological salt solution; FPSS, calcium-free PSS.

^8^Br-cGMP (100 *μ*M/l) changed the response of the arteries to ANG II after I/R in a manner similar to SNP (Fig. 3).

### Effects of the inhibitors L-NAME and ODQ

**Figure 4.**
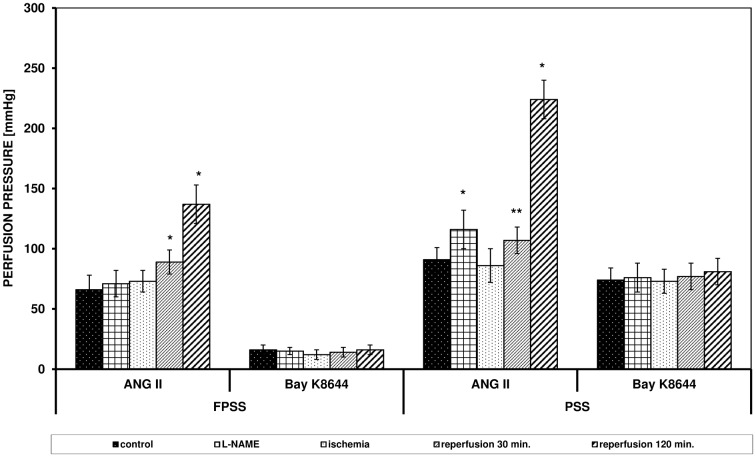
Effect of 60 min ischemia and 30 and 120 min of reperfusion on response of the arteries to ANG II (30 nM/l) and Bay K8644 (30 *μ*M/l) in the presence of L-NAME (300 *μ*M/l), in FPSS and PSS; (mean ± SE, n=12); ^*^P<0.0001 vs. control; ^**^0.05>P>0.0001 vs. control. ANG II, angiontensin II; L-NAME, L-NG-nitroarginine methyl ester; PSS, physiological salt solution; FPSS, calcium-free PSS.

**Figure 5.**
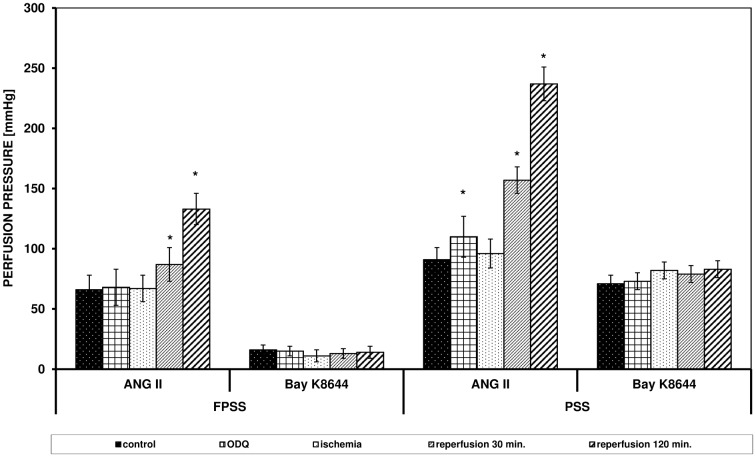
Effect of 60 min of ischemia and 30 and 120 min of reperfusion on response of the arteries to ANG II (30 nM/l) and Bay K8644 (30 *μ*M/L) in presence of ODQ (100 *μ*M/l), in FPSS and PSS; (mean ± SE, n=12); ^*^P<0.0001 vs. control. ANG II, angiotensin II; ODQ, an inhibitor of soluble cG; PSS, physiological salt solution; FPSS, calcium-free PSS.

L-NAME (an NOS inhibitor; 300 *μ*M/l) and ODQ (a cG inhibitor; 100 *μ*M/l) did not significantly influence the perfusion pressure triggered by ANG II with participation of an intracellular pool of Ca^2+^, but in PSS, an increased reaction was observed (91 vs. 116 mmHg in the presence of L-NAME, P= 0.0004; 91 vs. 110 mmHg in the presence of ODQ, P= 0.0022; Figs. 4 and 5).

The two inhibitors eliminated the inhibitory effect of ischemia on the response of the arteries triggered by ANG II and increased the contraction resultant from reperfusion in FPSS and PSS (Figs. 4 and 5). After ischemia, in the presence of L-NAME, the perfusion pressure in PSS was 86 mmHg (vs. 24 mmHg without the inhibitor, P<0.0001), after 30 min of reperfusion the pressure reached 107 mmHg (vs. 87 mmHg without the inhibitor, P= 0.0027). In PSS, following the addition of ODQ, the perfusion pressure after ischemia was 96 mmHg (vs. 24 mmHg without the inhibitor, P<0.0001) and after 30 min of reperfusion it was 157 mmHg (vs. 87 mmHg without the inhibitor, P<0.0001).

L-NAME and ODQ did not influence the action of Bay K8644.

## Discussion

Surgical procedures, including bypass grafting and transplantations, are associated with temporary ischemia in organs and their reperfusion, which results in local contraction of the smooth muscles (13,16). Elevated vascular muscle contractile tension following I/R triggers an excessive increase in calcium ion concentration as well as damage of the endothelial cells and the smooth muscles, which disturbs the balance between factors which stimulate the contraction and relaxation of the vessels, and may lead to total elimination of the blood flow (11,17).

In the present study, the effect of I/R on contraction triggered by ANG II (an agonist of metabotropic angiotensin receptor AT1) and Bay K8644 (an agonist of calcium channels) was analyzed. In order to establish the significance of calcium ions (from intracellular reserves and the extracellular fluid), the experiments were conducted in fluid without calcium ions (for evaluation of the significance of the intracellular pool) and in standard Krebs fluid, after emptying the cellular reserves of calcium (for evaluation of the significance of the extracellular pool). Then, the effects of signaling pathways associated with NO and cGMP on the aforementioned reactions were evaluated.

The presented results reveal that in the case of contraction triggered by ANG II, the two pools of calcium ions mediate the response, but the response in PSS was more intense, and Bay K8644 led to an increase of the perfusion pressure only in the presence of calcium ions entering the cell from outside, since the action of Bay K8644 results from the direct activation of dihydropyridinic calcium channels located in the cellular membrane. Similar effects were also observed in studies which evaluated human mesenteric arteries (18,19).

It is known that NO is a basic substance of endothelial origin, which triggers effects via cGMP (20,21). Cyclic nucleotides, such as cAMP and cGMP, act contrary to calcium ions in smooth muscles. By lowering the level of [Ca^2+^]_i_ and the smooth muscle sensitivity to calcium ions, these nucleotides cause the vessel to relax (22–24). Besides the modulation of the smooth muscle, the signaling cascade NO → cG → cGMP inhibits artery proliferation and prevents aggregation and inflammatory effects, which indicates the potential suitability of this pathway, and the opportunity for the use of drugs administered in circulatory diseases (25–28).

Results of assays evaluating the response of the arteries to ANG II following I/R revealed that ischemia reduces and reperfusion intensifies the action of this peptide. The results of previous experiments have indicated that inhibiting the effect of ischemia on the contraction of arteries is associated with the presence of endothelium, NO synthesis and cGMP activation. The experiments which were conducted on rat tail arteries with removed endothelium revealed no reduction in response to ANG II following ischemia. However, intensification of the contraction following reperfusion was observed under these conditions (21). Studies on human mesenteric arteries revealed a modulating effect of NO, thromboxane A2 and guanylate cyclase on the reactivity of the vessels following I/R (29,30). Finally, studies of the effects of catalase and aminotriazole on the contraction of the caudal artery in rats stimulated by ANG II after I/R revealed that an antioxidative system modulates the responses to ANG II, and reperfusion disturbs the balance between antioxidants and the production of ROS (31).

A series of experiments conducted on the influence of Bay K8644 revealed no effect of I/R on responses to direct activation of the dihydropyridinic calcium channels. Similarly, experiments using a depolarizing concentration of KCl revealed that ischemia does not influence the response of the arteries to KCl (27). It should be noted that in the present study SNP reduced the contraction triggered by Bay K8644 in PSS, but L-NAME and ODQ did not change the response mediated by the entry of calcium ions through channels located in the cellular membrane. Similar results have been achieved in experiments on human mesenteric arteries, and in addition, it has been revealed that an increasing concentration of acetylcholine reduces the contraction stimulated by Bay K8644, but does not change the reaction in the presence of L-NNA and ODQ (19).

In conclusion, I/R modulate the contraction of arteries triggered by ANG II, but do not influence the reactions induced by Bay K8644. Intra- and extracellular pools of calcium ions mediate the action of ANG II, but Bay K8644 stimulates contraction only with the participation of calcium ions entering the cell. Regulation of the vascular smooth muscle tone associated with the action of NO and cGMP is subject to modulation under conditions of I/R.
